# Multi-Sensor Device for Traceable Monitoring of Indoor Environmental Quality †

**DOI:** 10.3390/s24092893

**Published:** 2024-05-01

**Authors:** Virginia Isabella Fissore, Giuseppina Arcamone, Arianna Astolfi, Alberto Barbaro, Alessio Carullo, Pietro Chiavassa, Marina Clerico, Stefano Fantucci, Franco Fiori, Davide Gallione, Edoardo Giusto, Alice Lorenzati, Nicole Mastromatteo, Bartolomeo Montrucchio, Anna Pellegrino, Gabriele Piccablotto, Giuseppina Emma Puglisi, Gustavo Ramirez-Espinosa, Erica Raviola, Antonio Servetti, Louena Shtrepi

**Affiliations:** 1 Department of Energy, Politecnico di Torino, 10129 Turin, Italy; giuseppina.arcamone@polito.it (G.A.); arianna.astolfi@polito.it (A.A.); alice.lorenzati@polito.it (A.L.);; 2 Department of Electronics and Telecommunication, Politecnico di Torino, 10129 Turin, Italyalessio.carullo@polito.it (A.C.); franco.fiori@polito.it (F.F.); erica.raviola@polito.it (E.R.); 3 Department of Control and Computer Engineering, Politecnico di Torino, 10129 Turin, Italy; pietro.chiavassa@polito.it (P.C.); gustavo.ramirez@polito.it (G.R.-E.);; 4 Department of Environment, Land and Infrastructure Engineering, Politecnico di Torino, 10129 Turin, Italydavide.gallione@polito.it (D.G.); nicole.mastromatteo@polito.it (N.M.); 5 Department of Electrical and Information Technology Engineering, Università di Napoli Federico II, 80138 Naples, Italy; 6 LAMSA—Department of Architecture and Design, Politecnico di Torino, 10129 Turin, Italy; 7 Logistics and Sustainability Department, Campus Management, Politecnico di Torino, 10129 Turin, Italy; 8 Department of Electronics, Pontificia Universidad Javeriana, Bogotá 110231, Colombia

**Keywords:** indoor environmental quality, multi-sensor, metrological characterization, uncertainty, bare sensors

## Abstract

The Indoor Environmental Quality (IEQ) combines thermal, visual, acoustic, and air-quality conditions in indoor environments and affects occupants’ health, well-being, and comfort. Performing continuous monitoring to assess IEQ is increasingly proving to be important, also due to the large amount of time that people spend in closed spaces. In the present study, the design, development, and metrological characterization of a low-cost multi-sensor device is presented. The device is part of a wider system, hereafter referred to as PROMET&O (PROactive Monitoring for indoor EnvironmenTal quality & cOmfort), that also includes a questionnaire for the collection of occupants’ feedback on comfort perception and a dashboard to show end users all monitored data. The PROMET&O multi-sensor monitors the quality conditions of indoor environments thanks to a set of low-cost sensors that measure air temperature, relative humidity, illuminance, sound pressure level, carbon monoxide, carbon dioxide, nitrogen dioxide, particulate matter, volatile organic compounds, and formaldehyde. The device architecture is described, and the design criteria related to measurement requirements are highlighted. Particular attention is paid to the calibration of the device to ensure the metrological traceability of the measurements. Calibration procedures, based on the comparison to reference standards and following commonly employed or ad hoc developed technical procedures, were defined and applied to the bare sensors of air temperature and relative humidity, carbon dioxide, illuminance, sound pressure level, particulate matter, and formaldehyde. The next calibration phase in the laboratory will be aimed at analyzing the mutual influences of the assembled multi-sensor hardware components and refining the calibration functions.

## 1. Introduction

The indoor environment, in which people spend about 90% of their time, influences occupants’ health, well-being, comfort, and work productivity [1]. The four main aspects addressed when talking about Indoor Environmental Quality (IEQ) are thermal, visual, acoustic, and Indoor Air-Quality (IAQ) domains. Due to the implications between conditions inside a building and human beings, IEQ has become a highly investigated topic, and concerns about possible building effects on occupants’ illnesses have grown [2]. The literature review by Ghaffarianhoseini et al. [3] shows how IEQ parameters (e.g., temperature, humidity, ventilation, illuminance level, noise, and air quality) can be considered physical contributors to the raising of Sick Building Syndrome (SBS) symptoms, with consequences on well-being, comfort, and work productivity. This evidence makes of fundamental importance the assessment of indoor conditions and the IEQ cross-domain effects on occupants’ perception and behavior [4]. Studies have conducted in-field monitoring of IEQ parameters and collected occupants’ feedback to assess their perception of the influence of indoor conditions on their health, comfort, and work productivity in working environments [5]. Nevertheless, at present, a set of parameters and a standardized methodology for the IEQ evaluation have not been defined yet [6].

Guaranteeing healthy indoor environments has been a concern since the 1970s [7], but at first, standards and guidelines commonly considered one aspect of IEQ at a time [8]. National and international standards provide thresholds for thermal, acoustic, lighting, and air-quality parameters, e.g., standards ANSI/ASHRAE 55:2020 [9], ISO 7730:2005 [10], ISO 3382-3:2022 [11], NF S31-080 [12], and EN 12464-1:2021 [13]. On the other hand, standard EN 16798-1:2019 [14] specifies requirements related to parameters of thermal environment, indoor air quality, acoustics, and lighting, describing how to establish these parameters for building system design and energy performance calculations addressing indoor operative temperature, air speed, difference in CO_2_ concentration, total ventilation rate, A-weighted Sound Pressure Level (SPL), equivalent continuous SPL, daylight factor and average maintained illuminance, among the others.

The interest in the assessment of IEQ increased in the 1990s, and national building certification schemes were developed, becoming motivators for building owners to meet the performance guidelines [8,15,16]. The UK Building Research Establishment Environmental Assessment Method (BREEAM), introduced in 1990, is one of the first methods. Other building certification schemes are the US Leadership in Energy and Environmental Design (LEED) and the International WELL Building Institute’s WELL Building Standard. These certification schemes are an additional reference for the selection of parameters to be used for IEQ assessment in buildings.

Traditionally, IEQ monitoring required high investments with reduced data accessibility and interpretation to support occupants’ needs [17]. Presently, it is possible to deepen the research by performing intensive long-term monitoring campaigns thanks to the use of low-cost sensors within the IoT framework. The design of continuous IEQ monitoring systems through the implication of wireless sensor networks and cloud software platforms allows the continuous and simultaneous monitoring of thermal, acoustic, lighting, and air-quality domains [15,18]. To allow a wider evaluation of IEQ, the monitoring of more than one parameter through the combination of multiple sensors into a single-case unit is increasingly sought. Many multi-sensor devices are presented in the literature, e.g., the low-cost battery-powered device by Tiele et al. [18]; the Sentient Ambient Monitoring of Buildings in Australia (SAMBA) by Parkinson et al. [15]; the Intelligent Built Environment Monitor (IBEM) by Geng et al. [19].

Questions regarding low-cost sensor performance due to sensitivity, accuracy, and response time are still open. Low-cost sensors do not meet the performance requirements of regulatory equivalent reference devices, and a standardized procedure for their use, quality assurance tools, and calibration procedures are lacking [20].

Tiele et al. [18] tested the temperature, humidity, and carbon dioxide sensors against the CO210 Extech commercial system for one hour in a laboratory environment. Based on measurement results, they adjusted the temperature and CO_2_ readings by 1.9 °C and 70 ppm, respectively. Then, they did a baseline calibration of the device, putting it and the CO210 inside a sealed plastic enclosure and exposing it to zero air.

Parkinson et al. [21] performed the calibration in a controlled environmental chamber designed to suit the application in commercial offices, which means parameter measurement ranges typical of this kind of environment. The thermal sensors calibration was performed through a small-scale wind tunnel, whereas the calibration of indoor air-quality sensors was made through a sealed chamber where reference gases were supplied by means of an intake port. The calibration of the illuminance sensor consisted of the positioning of a dome on top of SAMBA with an RGB LED module (WS2812, Worldsemi-Dalingshan, China) mounted as a point-source. The reference device was the T10A, Konica Minolta. The microphone was calibrated, locating it with a reference SPL meter (Type 1; NL-52, Rion, Tokyo, Japan) near a monitor generating a noise signal in the frequency range of 100 Hz to 16 kHz.

Geng et al. [19] had their device calibrated in a testing chamber by the National Institute of Metrology and then tested it for two days through comparison with commercial sensors. The devices were put in five locations with different controlled conditions in a two-floor unoccupied office to ensure a wide parameter range during the test. They also implemented a self-calibration for CO_2_ and illuminance sensors through an algorithm that automatically compares the minimum measured value in a certain period with the baseline value (respectively, 400 ppm and 0 lx) and then removes the offset.

In this context, a multi-sensor device was designed for the monitoring of IEQ parameters as part of the system named PROMET&O (PROactive Monitoring for indoor EnvironmenTal quality & cOmfort) [22,23,24]. The main purpose of the project is the development of a system that comprises a multi-sensor device for the monitoring of IEQ parameters and a questionnaire to obtain occupants’ subjective feedback on their comfort perception and well-being. These data are collected, stored in a server, and then processed to calculate an index of thermal comfort, acoustic comfort, visual comfort, indoor air-quality perception, and an overall IEQ index. All these data are then displayed to the end user in an ad hoc developed dashboard. Furthermore, ten LEDs on the external case of the multi-sensor switch on or off depending on the IEQ index value calculated from the monitored parameters. The steps forward compared to current literature and to other multi-sensors already developed and distributed on the market are (i) the inclusion of sensors for the monitoring of all the IEQ domains; (ii) the collection of both subjective and objective data, thus allowing for the comparison on a dashboard of the perceived and calculated air quality, thermal, acoustic and visual comfort indexes; (iii) the reliability of the measured data by means of the implementation of a metrological confirmation process [25], which consists of identifying the requirements of the monitored parameters in terms of range and uncertainty and then characterizing each measurement chain to check its conformity with respect to the identified requirements. Regarding (iii), the main studies identified in the literature performed calibration directly with the assembled device. The calibration details of the assembled devices described in the recent literature are summarized below:

- Tiele et al. [18] performed the calibration in a laboratory for temperature, humidity, and CO_2_ sensors;
- Parkinson et al. [21] performed the calibration in a laboratory against reference devices over measurement ranges typical of offices;
- Geng et al. [19] had their device calibrated in a testing chamber by the National Institute of Metrology, and then they tested it through an in-field evaluation in a controlled office against commercial sensors.

In the end, they all had the device calibrated in a laboratory, and Geng et al. [19] also performed a second step test in an office environment. The PROMET&O project foresees a three-step calibration, which consists of (i) calibration of the bare sensors in the laboratory with the aim of obtaining the preliminary calibration functions, which are described in this work; (ii) calibration of the assembled multi-sensor in the laboratory, with the aim of analyzing the mutual influences of hardware components and to refine the calibration functions; (iii) in-field evaluation in a controlled office against laboratory-grade sensors, with the aim of assessing the uncertainty over the measurement ranges typical of offices.

Furthermore, this system is supposed to be implemented and connected to BACS (Building Automation and Control Systems) in buildings. For this reason, the reliability of monitored data is greatly required.

Requirements and results of the calibration procedures for the bare sensors of air temperature and relative humidity, illuminance, sound pressure level, carbon dioxide, particulate matter, and formaldehyde are presented.

## 2. Materials and Methods

The following paragraphs deal with (i) IEQ parameters selection and corresponding standards requirements; (ii) architecture of the system and 3-D arrangement; (iii) employed sensors and nominal characteristics; (iv) data collection and visualization; and (v) calibration requirements.

### 2.1. IEQ Parameter Selection and Corresponding Standards Requirements

A universally recognized group of parameters and indexes for IEQ assessment is not available yet [26]. Thus, a literature review of the main parameters to be monitored was carried out (see [27]) to identify the sensors to be included in the developed device. Based on that study and sensor characteristics, accuracy, dimensions, cost, and market availability, the parameters to be monitored were chosen. Concerning the thermal domain, indoor air temperature and relative humidity resulted in the most commonly monitored parameters in the reviewed studies (100% and 95%, respectively). The main reference standards consulted for the thermal domain are EN 16798-1:2019 [14], ANSI/ASHRAE 55:2020 [9], and ISO 7730:2005 [10]. Concerning the visual domain, illuminance values are monitored through the PROMET&O multi-sensor since it was the parameter assessed in all the analyzed studies due to the higher complexity in monitoring other parameters. Standards EN 16798-1:2019 [14] and EN 12464-1:2021 [13] were considered to be references. Concerning the acoustic domain, SPL is monitored, and A-weighted SPL is considered, with reference to EN 16798-1:2019 [14], ISO 3382-3:2022 [11], and NF S31-080 [12]. Statistical values (L_10_ and L_90_) are also determined. Concerning IAQ, many parameters are monitored with the further aim of determining the ones that better correlate with comfort perception. Carbon dioxide, carbon monoxide, particulate matter, formaldehyde, volatile organic compounds, and nitrogen dioxide were selected. Threshold values for these parameters are listed in the standard EN 16798-1:2019 [14] with reference to the WHO (World Health Organization) guidelines. Table 1 shows the parameters monitored by the multi-sensor and their thresholds for office environments defined by the standards.

### 2.2. Architecture of the System and 3-D Arrangement

**Figure 1:**
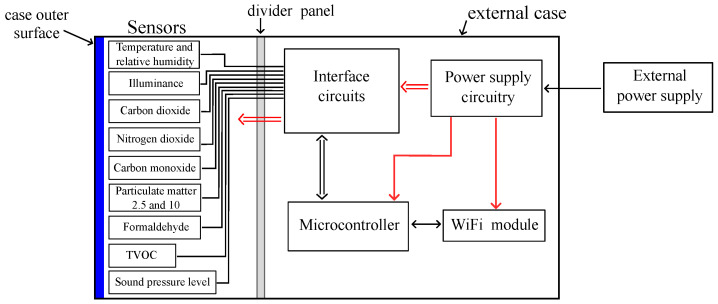
Block scheme of the PROMET&O multi-sensor.

Following the requirements stated in Section 2.1, the PROMET&O device was designed according to the block scheme that is shown in Figure 1. The required physical quantities are detected through low-cost sensors, whose output signals are connected to the onboard microcontroller through interface circuitry. More precisely, such interface circuits are required to properly bias the sensors and to adapt the output signals of the CO and NO_2_ sensors to the input range of the Analog-to-Digital Converter (ADC) of the microcontroller. The microcontroller periodically acquires the data from all sensors and transmits them to a cloud server, exploiting a WiFi module with the aim of storing, visualizing, and post-processing. Two DC-DC converters supply all the sub-blocks of the system from an external power supply.

Regarding the firmware running on the microcontroller, the firmware has been conceived to sample the output signal of each sensor with a specific sampling frequency and implement the corresponding calibration function. Once an amount of time defined as report time has elapsed, statistical calculations are performed on the data collected from the sensors, and, finally, they are transferred to the WiFi module to be sent to the cloud.

**Figure 2:**
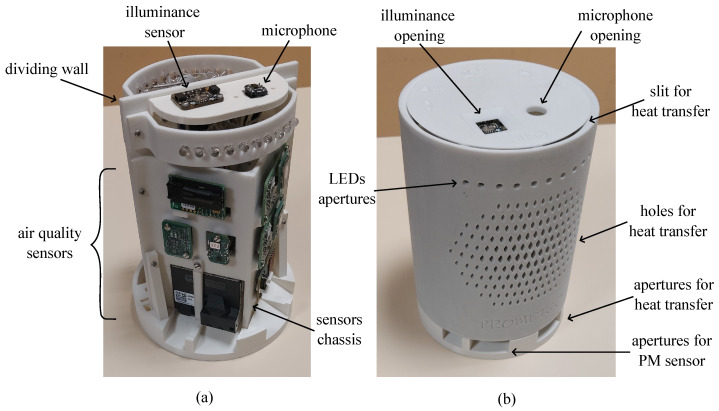
Photograph of (**a**) the internal parts and (**b**) the assembled PROMET&O multi-sensor.

The sub-blocks of the system have been arranged as shown in Figure 2a, where the internal parts of the multi-sensor are visible. Temperature, relative humidity, and air-quality sensors have been placed on a vertical V-shaped mount to keep their sensitive elements as close as possible to the internal surface of the case. In such a way, a representative assessment of the physical quantities monitored by the device can be achieved. Conversely, the illuminance sensor and the microphone have been positioned on a different support on top of the structure to affect their spatial responsivity as little as possible. Light Emitting Diodes (LEDs) have also been implemented as two clusters of ten units per side to provide end users with visual feedback regarding the monitored environment. All the remainder sub-blocks shown in Figure 1, i.e., the interface circuits, the microcontroller, the power supply, and the WiFi module, have been placed on the opposite side of the structure, thus being not visible in Figure 2a. Finally, a dividing wall thermally separates the exploited sensors from potential sources of heat, which can be microcontrollers, WiFi modules, and power supply circuitry.

The assembled PROMET&O device is shown in Figure 2b. The 3-D printed external case is cylindrical, 188 mm in height, and 130 mm in diameter. In correspondence with the sensitive elements of the light sensor and microphone, two openings on the top cover have been realized. The cylindrical surface of the external case is characterized by a series of holes to facilitate heat transfer to the surrounding environment. The holes visible in Figure 2b have also been realized on the other side of the structure. A total of four apertures have been realized on the bottom. Referring to Figure 2b, the two holes on the left provide the particulate matter sensor with direct access to external air. More precisely, a first hole is required for the air inlet and a second one for the air outlet. The remainder of the apertures on the bottom have been realized to further help in the dispersion of heat. For the same reason, a small slit separates the top cover from the case.

### 2.3. Employed Sensors and Nominal Characteristics

The main parameters of the selected commercial sensors are discussed here. More precisely, for each selected sensor, the corresponding measurement range (SMR), declared uncertainty (DU) value, and range of declared uncertainty (RDU) have been reported in Table 2. The uncertainty declared by manufacturers should be compared to the required uncertainty (RU). Uncertainties highlighted in bold refer to sensors that require a metrological characterization, as their declared uncertainties do not meet the corresponding standard requirements.

Sensors were chosen to take into consideration measurement range and uncertainty in the first place, as well as cost, physical dimensions, response time, and power consumption. Concerning the choice of temperature and relative humidity sensors, very small devices (around some cubic millimeters) can be found on the market that measure both these quantities and achieve accuracies up to a tenth of a degree. Regarding the illuminance sensor, the spectral response should be considered to be good, and it should represent the human eye photopic curve as closely as possible. The selected sensor is based on a photodiode.

A Micro-Electro-Mechanical Systems (MEMS) microphone has been selected for sound pressure level measurements. This kind of sensor detects acoustic waves through a capacitive sensing element, where a fixed backplate is present together with a movable diaphragm; the two of them are separated by an air gap. When an acoustic pressure is applied, the diaphragm moves, and the measured capacitance varies accordingly [31].

The CO_2_ sensor is based on a Non-Dispersive Infrared (NDIR) cell where a beam from an IR source is directed through a chamber toward a detector; absorption of specific wavelengths occurs depending on the gas present inside the cell. At the end of the chamber, a detector measures the attenuation of the wavelength of interest and, consequently, the concentration of the gas can be derived [32].

Particulate matter sensor relies on the Optical Particle Counter (OPC), which is based on laser scattering. Air enters inside a measurement cell and is hit by a laser beam characterized by a wavelength of 660 nm [33]. Light is scattered with different intensity towards a photodetector.

Carbon monoxide, nitrogen dioxide, and formaldehyde concentrations are measured through electrochemical cells. These kinds of sensors are based on a chemical reaction, and they provide an output current that is proportional to the concentration of the gas of interest.

### 2.4. Data Collection and Visualization

A web-based interactive user interface has been implemented for real-time monitoring and graphical data analysis of the multi-sensor measurements that are collected in a cloud database system. The software infrastructure of the backend system is based on a multiservice architecture that relies on docker containers to allow seamless scalability and customization. The primary services that compose the software platform are an MQTT broker (mosquito), a python MQTT client, a database server (MySql), python schedulers, a Web-API (Flask), a data visualization tool (Grafana) and a web application for the end user interaction. Additional network and security services were configured to verify and personalize access to the resources via an authentication proxy (based on nginx) and an identity provider (keycloak), which allow integration and single sign-on with third-party domains.

The multi-sensor device sends collected measurements to the mosquito MQTT broker using the MQTT protocol. The messages are received by the MQTT client, which stores their content inside the database. On a regular basis, custom routines are scheduled on the database to process the stored measurements and enrich the stored data—eventually by accessing external resources such as weather services—with time aggregations of different granularity (real-time, three hours, one day, three days, one week, one month). Furthermore, a percentage value describing the quality of the environment is calculated for each domain (i.e., thermal, visual, acoustic, IAQ) and the overall IEQ through specific algorithms based on the principle of compliance in time of the monitored parameters.

The Web-API provides an interface to access the information stored in the database, which is queried by Grafana to generate multiple easy-to-customize data visualizations that update in real time. The web application embeds the Grafana visualizations and provides user-friendly real-time access to the measured data and calculated indexes. It also allows the user to select multiple graphs and compare them side-by-side to analyze the trend of the measured quantities over multiple time intervals.

Through the interface, users can also answer a questionnaire to provide feedback on their comfort perception related to the thermal, acoustic, visual, and air-quality domains after a first evaluation of the overall IEQ of the environment in which they are placed. The questionnaire was developed and validated [24] with the aim of providing a non-intrusive and quick-to-fill-out system of feedback collection.

### 2.5. Calibration Requirements

Low-cost sensors are emerging as one of the alternatives for monitoring environmental variables, in compliance with regulations such as Directive 2008/50/EC on Ambient Air Quality and Cleaner Air for Europe [34]. This directive establishes values for the assessment of various pollutants present in the air, as well as Data Quality Objectives (DQO) for their measurement based on uncertainty. Building upon this, the CEN/TS 17660-1:2021 standard [35] defines evaluation parameters for air-quality sensors measuring gaseous pollutants ($O_{3}$, NO/${\mathrm{NO}}_{2}$/${\mathrm{NO}}_{x}$, CO, SO_2_, and benzene) and classifies them based on non-regulatory measurements. Additionally, this standard provides guidance for testing ${\mathrm{CO}}_{2}$ sensors, although ${\mathrm{CO}}_{2}$ is not listed in Directive 2008/50/EC. In the case of particulate matter, there is currently no equivalent standard for low-cost sensors, but some proposals are presented in [36].

In this project, according to the metrological characteristics of the selected sensors and the required uncertainty by international standards and building certification schemes, two different scenarios are considered for the calibration of the measurement chain of each sensor. The former consists of a verification procedure of the whole measurement chain against a reference standard to evaluate the error of the chain and verify if it complies with the target maximum admitted error. It is applied whenever the uncertainty stated by the sensor manufacturer meets the requirements. The latter consists of modifying the calibration function of the measurement chain by means of a metrological characterization with a reference standard. It is applied whenever the sensor specifications do not meet the uncertainty requirements. This information is summarized in Table 2. The two different conditions are highlighted in the column “Parameter”: italic text for the first condition (the sensor is nominally able to meet the uncertainty requirement) and bold text for the second condition (the sensor requires a suitable characterization).

It is to be noted that in this first stage of development of the multi-sensor, only the air temperature, relative humidity, illuminance, sound pressure level, carbon dioxide, particulate matter, and formaldehyde sensors have been tested.

## 3. Calibration Procedures

The following paragraphs describe the calibration procedures designed and adopted for the sensors of air temperature and relative humidity, illuminance, sound pressure level, carbon dioxide, particulate matter, and formaldehyde. The presented measurements were performed with bare sensors. The same measurements are planned to be performed with the entirely assembled multi-sensor to evaluate its performance and the mutual interference of the hardware components.

### 3.1. Air Temperature and Relative Humidity

The air temperature and relative humidity sensor (Sensirion SHT41 - Sensirion, Stäfa, Switzerland) was verified inside a climatic chamber ACS Angelantoni DM340. For the temperature test, the reference measurement chain included a platinum resistance thermometer (Pt100) connected to a digital multimeter, which ensures an uncertainty of ±0.1 °C. The humidity test was performed against a reference hygrometer (Rotronic Hygropalm HP32) with an uncertainty of ±1%RH in the range (20÷80)%RH.

**Figure 3:**
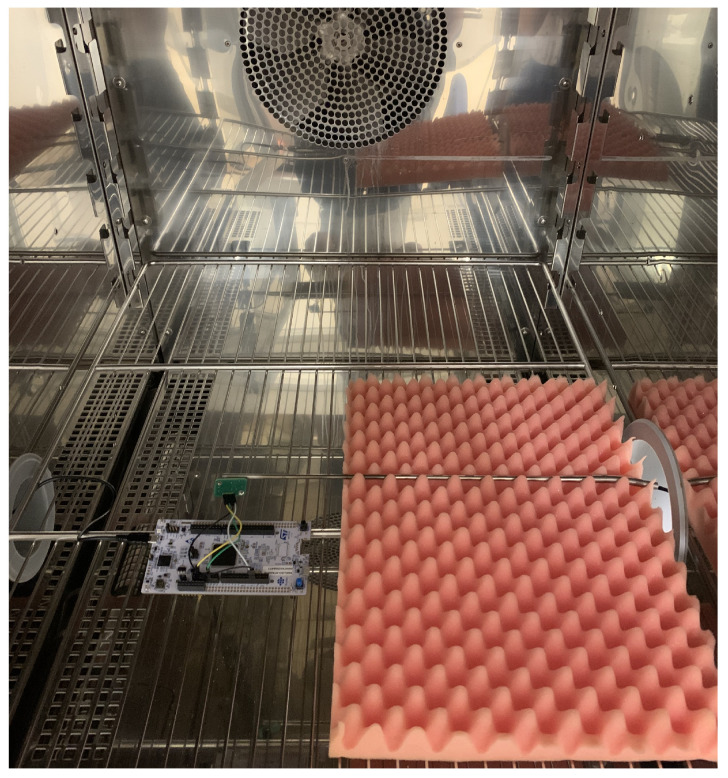
Measurements set-up for the verification of the temperature sensor (Sensirion SHT41).

For the air temperature measurements, both the sensor and the reference Pt100 were placed inside the climatic chamber, and the set points of 10 °C, 20 °C and 30°C were tested since they represent the most suitable conditions for the sensor range of use. The sensor was connected to the microcontroller, and this was connected to a PC. The reference Pt 100 sampled one data point every 30 s. Thus, the data sampled every second through the sensor were averaged over 30 s, for a total of about 12 min for each temperature setting. The measurement set-up inside the climatic chamber is shown in Figure 3.

The relative humidity sensor verification procedure was performed in the test points 30%RH, 40%RH, 50%RH, 60%RH, 70%RH and 80%RH at a constant temperature of 23 °C. Once the required conditions were achieved, the sensor sampled one data point per second and then averaged the values over 30 s, resulting in one data point every 30 s. The reference hygrometer sampled one data point every 30 s. Each test, with the different configurations, lasted about 30 min.

### 3.2. Illuminance

**Figure 4:**
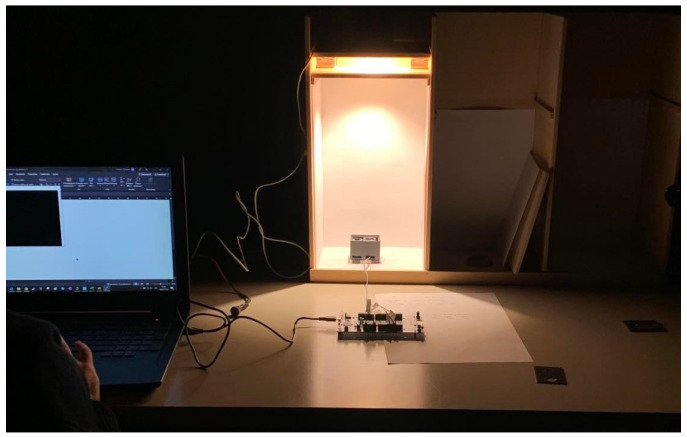
Measurements set-up for the calibration of the illuminance sensor. Taken from [22].

The low-cost illuminance sensor (Vishay VEML7700 - Vishay Intertechnology, Inc., Malvern, PA, USA) was calibrated by comparison to a reference instrument (PRC Radiolux 111 luxmeter) equipped with a photometric head certified in class B according to DIN 5032-7 [37]. The tests were performed inside a completely dark room, under stable thermal conditions, and through a test box containing the LED source (see Figure 4).

The calibration method consisted of measuring illuminance under the stable light source, first through the reference instrument and then through the sensor under calibration. The distance from the light source to the sensitive area was maintained unvaried during all the tests, ensuring a uniform light distribution on the measuring plane. The correct positioning of the sensitive area under the light source was checked through a laser beam pointer. Field experiments were conducted over several days to test multiple configurations. The following test conditions were adopted:

- test of the spectral response of the sensor using 3 LEDs with different correlated color temperatures and spectra (2700 K, 4000 K, 5700 K, warm white, neutral white, and cool white, respectively);
- test with illuminance values between 2.5 lux and 3500 lux, obtained by dimming each LED;
- test of the response at different angles of light incidence using two supports inclined at 30° and 60°;
- test with and without a scaled device case with an upper window opening of different sizes and thicknesses (see Figure 4).

The data were collected through the reference instrument directly on an Excel spreadsheet, previously programmed to report one data point per second for a total of 30 data elements, then averaged. The low-cost sensor Vishay VEML7700 acquired one data per second for 30 s, too.

### 3.3. Sound Pressure Level

The MEMS microphone (ST-IMP34DT05—STMicroelectronics, Geneva, Switzerland) is verified by comparison to a reference class-1 microphone (NTI MC230A—NTi Audio, Schaan, Liechtenstein) in the anechoic chamber of Politecnico di Torino, which has a volume of 144 m^3^ and a background noise level of 17.3 dB(A). The NTI microphone was calibrated against sound pressure calibrator B&K, assuming 94 dB at 1 kHz. Both microphones were exposed to the same free-field sound pressure, and the free-field sensitivity of the microphone under-test was evaluated with respect to the free-field sensitivity of the reference microphone. The source B&K Sound Power Source type 4205, thanks to a metallic scaffold, allowed to place the MEMS and the NTI at the same position and at a distance of 7.5 cm from the loudspeaker, which emitted a stationary white noise both broadband (100 Hz–10 kHz) and narrowband for each octave band from 125 Hz to 4 kHz. The sound pressure level at the microphone position was higher than the background noise level of more than 30 dB for each frequency band. The acoustic centers or reference points of both microphones were positioned at the same measurement point with the specified angle of incidence of 90°. Since the manufacturer of the MEMS microphone states an uncertainty of ±3dB, which is higher than the required uncertainty (±0.5dB), it was chosen to perform the metrological characterization of the MEMS microphone.

The sensitivity was obtained from the comparison of the SPL measured by the NTI microphone and the MEMS microphone when the source was emitting the broadband white noise at 55 dB, 65 dB, 75 dB, 85 dB, and 95 dB.

During the multi-sensor design phase, particular attention was given to the placement of hardware components to minimize mutual influences. As far as the microphone is concerned, it has been positioned on the top of the PROMET&O device, as shown in Figure 2, to distance it as much as possible from the particulate matter sensor, which is the only hardware component equipped with a fan. The microphone inlet was oriented upwards, ensuring that the fan noise, which is approximately 24 dB(A) at 0.2 m as declared by the manufacturer, is expected to be negligible compared to typical office noise levels. Additionally, the noisier fan auto-cleaning feature will be scheduled for nighttime operation to prevent interference with SPL monitoring.

### 3.4. Carbon Dioxide

**Figure 5:**
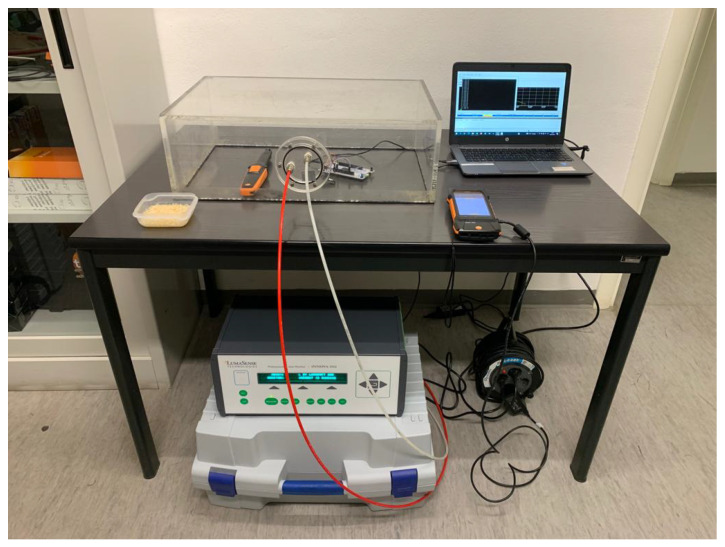
Measurements set-up for the calibration of the CO_2_ sensor. Taken from [22].

The verification procedure for the CO_2_ low-cost sensor (Sensirion SCD30—Sensirion, Stäfa, Switzerland) was performed by comparison to the Photoacoustic Gas Monitor—Innova 1512, which provides a relative uncertainty of ±2.5% in the measurement range of interest. The test was conducted inside an unoccupied office room. The low-cost sensor was arranged inside a polymethyl methacrylate (PMMA) box connected to the Photoacoustic Gas Monitor via two polyamide 11 (PA 11) hoses (see Figure 5). The red hose injects air with known ${\mathrm{CO}}_{2}$ concentration into the box, while the white hose extracts air from the box and leads it to the gas tracer for analysis.

The chosen sample integration time was 5 s, while the sampling time was one data/s for the instruments.

Four test conditions were defined:

- Baseline (approximately 500 ppm)
- 1500 ppm
- 2000 ppm
- 2500 ppm

The first one was achieved by opening the windows and the box for a few minutes, then closing the box, and measurements started, lasting about one hour. The second, third, and fourth concentrations were reached by injecting ${\mathrm{CO}}_{2}$ into the box by steps through an air-inflated balloon. Silica gel was introduced inside the balloon to control the relative humidity. The tests lasted a few days to further analyze the sensor performance with CO_2_ concentration decay.

### 3.5. Particulate Matter

The calibration of the particulate matter sensor (Sensirion—SEN54, PM2.5, and PM10) was performed by comparison with the reference instrument APM-2, a certified air pollution monitor designed for continuous real-time monitoring of PM2.5 and PM10 concentrations. It is based on nephelometry as an alternative method to the usual gravimetric approach. Certified by TÜV Rheinland and conforming to MCERTS standards, the photometric method using scattered light has undergone qualification tests for the fractions PM10 and PM2.5. The measurement method is based on a highly sensitive scattered light sensor with a measurement range of $\left(0÷1000\right)\;\mu g/m^{3}$. The wavelength of 650 nm is sensitive to particle sizes between 0.5 μm and 1 μm. To compensate for the lack of sensitivity in PM10 measurements, the APM-2 uses a virtual impactor to select PM10 particles.

Measurements were carried out in a closed room located on the third floor of a building within the Politecnico di Torino. The room was not accessible to any person during the measurement period, and the door was kept closed. The instrument APM-2 was positioned above a desk, and the sensor was placed next to it. Before starting the measurement, the APM-2 was cleaned, and the filter was controlled to check the proper functioning. The test lasted about two hours, and the sampling rate of the APM-2 was two minutes, switching automatically from PM2.5 to PM10. The low-cost sensor sampled one data point/minute.

### 3.6. Formaldehyde

Formaldehyde (CH_2_O) is a transparent toxic gas with a pungent odor, and the WHO stipulates that the indoor formaldehyde content should not be higher than 0.08 ppm [38]. An electrochemical sensor was installed in the PROMET&O multi-sensor to measure this parameter. This typology of the sensor can have high selectivity and accuracy [39]. However, crafting sensors capable of accurately measuring formaldehyde levels at low concentrations has proven to be complex. Numerous factors influence formaldehyde concentration indoors, resulting in fluctuations in its emission. Apart from material properties, it is also correlated with indoor temperature, humidity, and ventilation rate. The signals of electrochemical sensors can be influenced by temperature and relative humidity [40,41]. The Sensirion SFA30 formaldehyde sensor was calibrated by comparing its readings to those of the Formaldemeter htV-M, manufactured by PPM Technology. The htV-M device is specifically designed for precise monitoring of low levels of indoor pollutant gases, with a focus on formaldehyde vapors, across various temperature and humidity conditions. It can accurately measure formaldehyde concentrations in both parts per million (ppm) and milligrams per cubic meter (mg/m^3^). The Formaldemeter htV-M adheres to ISO 9001:2015 [42] quality standards and CE regulations, ensuring its reliability and accuracy in measurements [43]. Specific filters were added to the instrument to filter out phenols intentionally, ensuring that only formaldehyde was measured accurately. These filters were directly installed at the instrument’s sampling point. The formaldehyde sensor was positioned close to the calibrated htV-M formaldemeter, near its intake port. The measurements took place in a secured room located on the third floor of a building in Politecnico di Torino. Throughout the measurement, the temperature and humidity remained stable. The formaldemeter was programmed to sample data every 15 min, while the low-cost sensor collected data every minute. The measurements lasted for 150 min.

## 4. Results

Experimental results are here reported that refer to the verification or the calibration of the measurement chains embedded in the PROMET&O device. According to the considerations of the previous section, verification was performed for the temperature and relative humidity sensors, while the measurement chains of illuminance, sound pressure level, and carbon dioxide required a metrological characterization. Regarding the particulate matter and formaldehyde sensors, preliminary results are available that refers to a baseline condition that does not allow a full verification to be assessed.

### 4.1. Air Temperature and Relative Humidity

**Figure 6:**
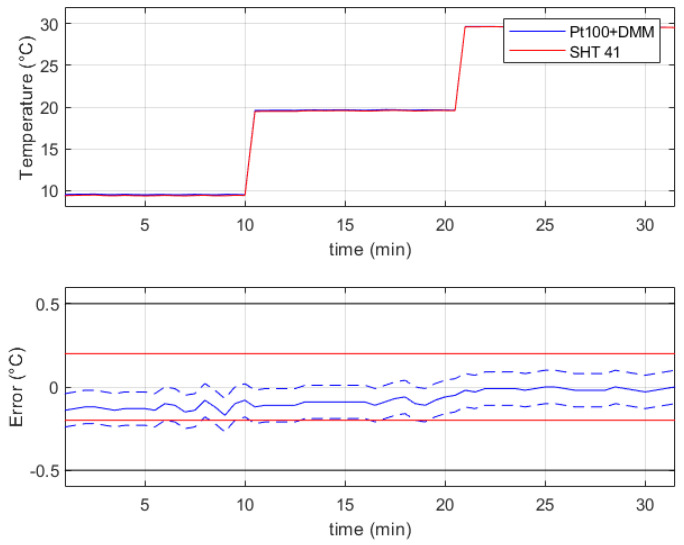
Experimental results related to the verification of the sensor Sensirion SHT41 in the temperature range from 10 °C to 30 °C. In the bottom chart is represented the measurement error *E* (continuous blue line), the error *E* summed to the expanded uncertainty U(E) of the error (couple of dashed blue lines), the stated uncertainty (couple of continuous red lines), and the required uncertainty (couple of continuous black lines).

The results that refer to the verification of the temperature sensor Sensirion SHT41 in the range from 10 °C to 30 °C are summarized in Figure 6, where the top chart shows the reference values (blue line) and the measurements of the device under verification (red line). The bottom chart of the same figure reports the measurement error *E* (continuous blue line) as the difference between the indications of the sensor SHT41 and the reference values, while the couple of dashed blue lines represent the error *E* summed to the expanded uncertainty U(E) of the error. One should note that the compliance of the sensor under verification to its stated uncertainty (a couple of continuous red lines) cannot be stated with high reliability since the quantity E−U(E) is lower than the lower tolerance of the sensor. However, the device under verification passed the test with respect to the required uncertainty (a couple of continuous black lines) that is ±0.5 °C.

The humidity verification was performed in the actual test points 38.6%RH, 47.8%RH, 56.8%RH, 67.1%RH, 77.4%RH and 86.7%RH with a temperature in the range from 22.5 °C to 24 °C. The error with respect to the reference hygrometer was lower than 2%RH with maximum deviations of −1.2%RH @ 86.7%RH and +1.8%RH @ 38.6%RH. Also, in this case, the compliance of the sensor under verification to its stated uncertainty (±1.8%RH) cannot be reliably assessed since the error uncertainty U(E)=1%RH, but it can be considered suitable with respect to the required uncertainty, which is ±5%RH.

### 4.2. Illuminance

**Figure 7:**
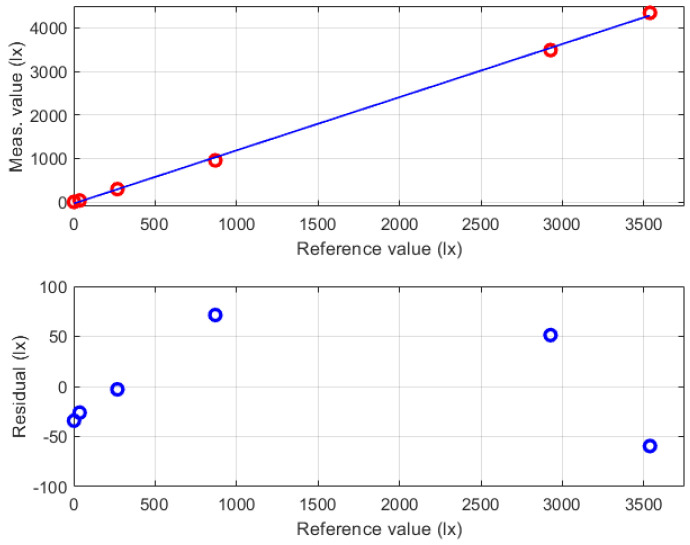
Characterization results for the illuminance measurement chain obtained using the LED with the correlated color temperature of 2700 K. In the top chart the red circles represent the couples of reference and measured values, while the blue line is the linear calibration function. The bottom chart reports the residual fitting errors (blue circles). Taken from [22].

For the measurement chain of the illuminance $E_{v}$, the manufacturer states a maximum relative admitted error of ±15%, which is larger than the required relative uncertainty of ±5%. For this reason, a preliminary characterization of the illuminance measurement chain was performed to identify its calibration function. Such a characterization has been performed using the results provided by the LED with the correlated color temperature of 2700 K (warm white), which exhibits a spectral response with a maximum sensitivity that is close to the maximum of the photopic human sensitivity V(λ). The obtained results are summarized in Figure 7, where the red circles in the top chart are the couples of reference and measured values, while the blue line is the linear calibration function (intercept –35 lx; slope 1.22 lx/lx) that was identified through the minimization of the root square sum of the difference between the function and the experimental values. In the same figure, the bottom chart reports the residual fitting errors, which show a negligible mean value and a root mean square error of about 50lx. Combining this contribution to the uncertainty specifications of the reference luxmeter, the expected uncertainty of the adjusted illuminance chain was evaluated, which can be expressed at a confidence level of 95% as:

(1) $$U_{\mathrm{adj}}\left(E_{v}\right)=\left(60+4\%\;measured\;value\right)\;\mathrm{lx}$$

**Figure 8:**
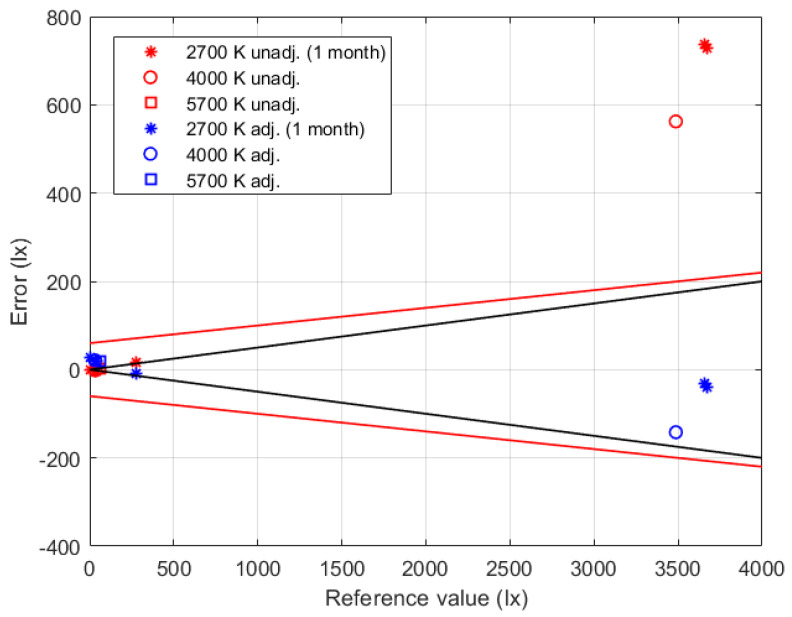
Verification results obtained for the illuminance measurement chain using its indications without any correction (unadjusted, red symbols) and using the identified calibration function (adjusted, blue symbols). The couple of black lines represent the maximum admitted error.

The calibrated illuminance measurement chain was then verified against the same reference device using all the available LEDs: the two devices with correlated color temperatures of 4000 K and 5700 K, which exhibit the maximum sensitivity of spectral response in the violet region of visible light, and the LED at 2700 K after one month from the characterization. Figure 8 summarizes the obtained results, where the red symbols refer to the errors obtained using the unadjusted indications, while the blue symbols are the errors that result from the adjusted indications that are obtained by the implementation of the identified calibration function. The couple of continuous red lines in the same figure refers to the expanded measurement uncertainty of the characterized chain, which is expressed by Equation (1). The effectiveness of the proposed characterization procedure is confirmed by measurement errors that are lower than the expected uncertainty (blue symbols within the region delimited by the couple of red lines). According to the described procedure, the verification results also allowed the spectral response of the sensor (LEDs with different correlated color temperatures) and the medium-term stability (results after one month from the characterization) to be taken into account. On the contrary, the unadjusted results (red symbols) show very large errors that also exceed the uncertainty stated by the manufacturer (±15% of the measured value) for illuminance values higher than 3500lx.

The couple of black lines in Figure 8 represent the maximum admitted error (±5% of the measured value) according to the measurement requirements for IEQ monitoring applications. One should note that the expanded uncertainty of the characterized chain slightly exceeds the target uncertainty; however, the obtained results can be considered a good trade-off between performance and cost of the developed illuminance measurement system.

### 4.3. Sound Pressure Level

**Figure 9:**
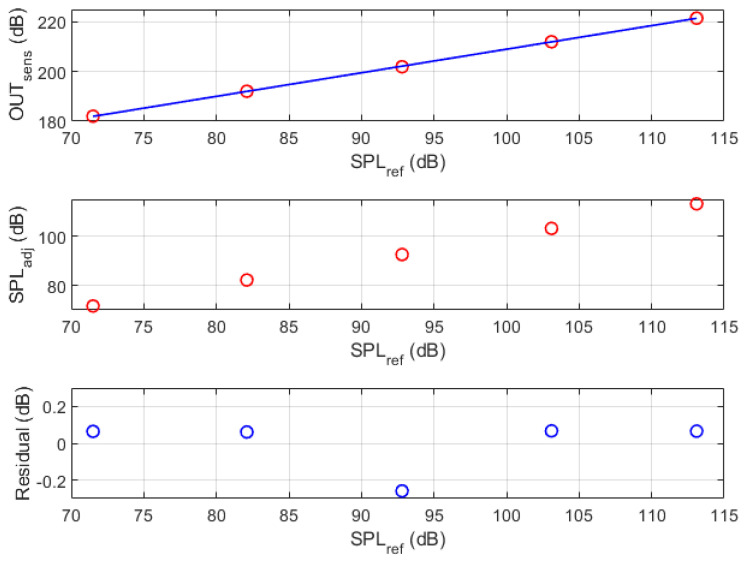
Characterization results for the SPL measurement chain. In the top chart the blue line represents the identified linear calibration function. The middle chart shows the SPL values (red circles) obtained by processing the output of the MEMS microphone with the identified calibration function, while the bottom chart reports the residuals between the experimental values and the fitted function (blue circles).

The sensitivity of the MEMS microphone that is embedded in the PROMET&O device is stated with an uncertainty of ±3dB, thus requiring a preliminary characterization to meet the target uncertainty of ±0.5dB. According to the procedure described in Section 3.3, the MEMS microphone was characterized using a broadband (100Hz÷10kHz) stationary white noise in the SPL range from about 75dB to about 113dB on the plane of the reference microphone. The results that relate the reference measurement to the raw data at the output of the device under calibration are shown in the top chart of Figure 9, where the blue line represents the identified linear calibration function (intercept 114.1 dB; slope 0.95 dB/dB). The middle chart of the same figure shows the SPL values that are obtained by processing the output of the MEMS microphone with the identified calibration function, while the bottom chart reports the residuals between the experimental values and the fitted function (negligible mean value and root mean square error of about 0.15dB).

**Figure 10:**
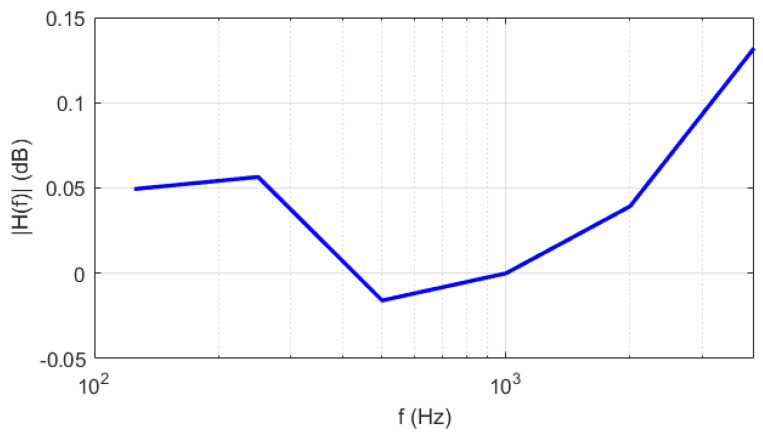
Frequency response of the MEMS microphone in the frequency range (125÷4000)Hz.

The results corresponding to the narrowband stationary white noise allowed the frequency response of the MEMS microphone to be evaluated for each octave band from 125Hz to 4kHz. Figure 10 shows the amplitude of the frequency response of the MEMS microphone, which exhibited a flatness of about 0.15dB in the investigated frequency range.

According to the obtained results and considering the uncertainty specifications of the reference class-1 microphone, an expanded uncertainty of 0.6dB is assigned to the measurement chain of the parameter SPL, which can be considered suitable for IEQ monitoring applications.

### 4.4. Carbon Dioxide

**Figure 11:**
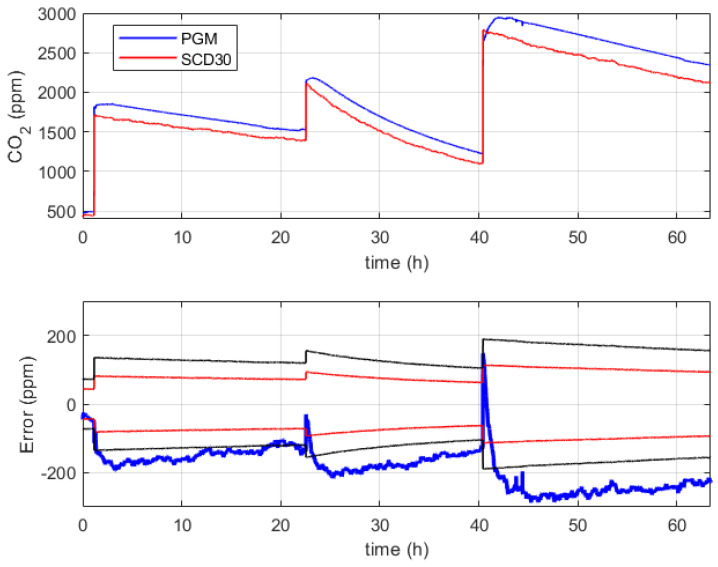
Verification results obtained for the ${\mathrm{CO}}_{2}$ concentration measurement chain. The bottom chart shows the measurement error (thick blue line), the stated uncertainty specifications (couple of red lines) and the target uncertainty (couple of black lines).

According to the procedure described in Section 3.4, the ${\mathrm{CO}}_{2}$ concentration sensor Sensirion SCD30 has been verified against the reference device PGM-Innova 1512 in the range from about 500 ppm to 3000 ppm. The verification results are reported in Figure 11, where the top chart shows the measurements of the reference device (blue line) and sensor under verification (red line), while the bottom chart shows the corresponding measurement error (thick blue line).

**Figure 12:**
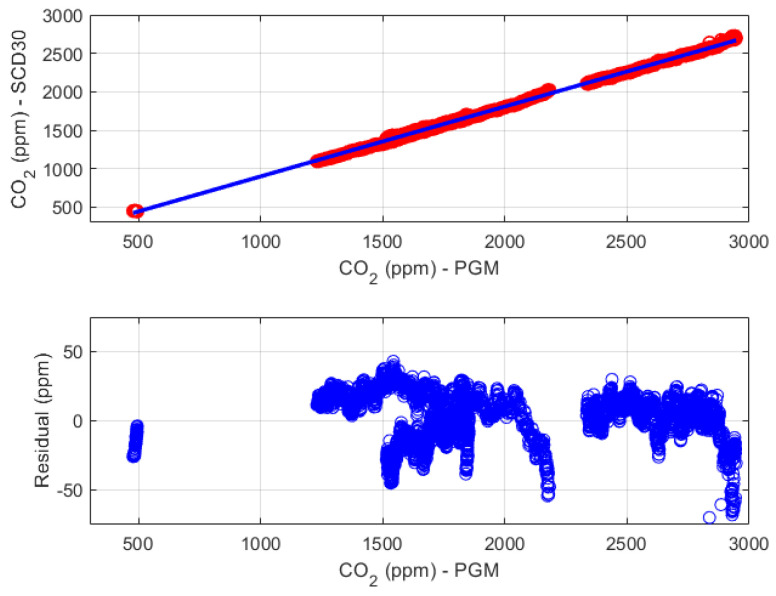
Characterization results for the ${\mathrm{CO}}_{2}$ concentration measurement chain. The top chart shows the linear calibration function (blue line), while the bottom chart shows the fitting residuals.

The main outcome of the experiment is the non-compliance of the device SCD30 with respect to both its stated uncertainty specifications (a couple of red lines) and the target uncertainty (a couple of black lines). As can be observed in Figure 11, the device SCD30 suffers from a large gain error since the difference with respect to the reference device increases as the input value increases. For this reason, the available experimental data, except for transients, has been used to identify a linear calibration function (intercept −11 ppm; slope 0.91 ppm/ppm), which is shown in the top chart of Figure 12. The bottom chart of the same figure shows the fitting residuals, which are characterized by an almost zero mean value and a root mean square error of about 18 ppm.

### 4.5. Particulate Matter

**Figure 13:**
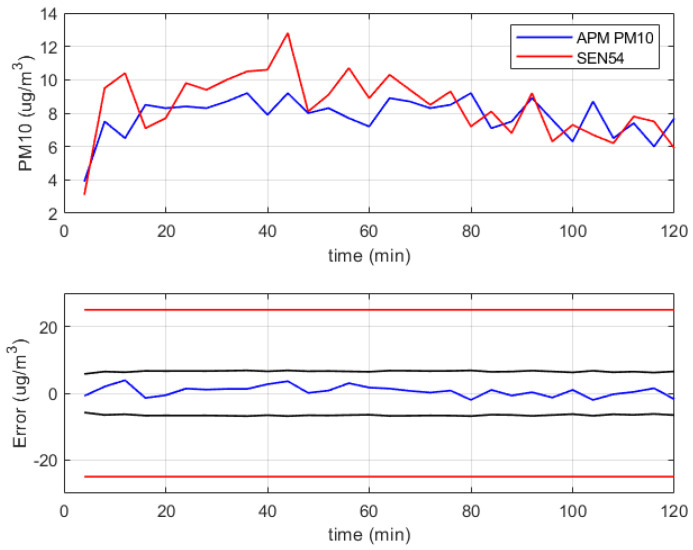
Experimental results related to the verification of the sensor Sensirion SEN54 for concentration of PM10 close to $10\;\mu g/m^{3}$. In the bottom chart are reported the measurement error of the device under verification with respect to the reference instrument (blue line), the stated specifications (couple of red lines), and the required uncertainty (couple of black lines).

Preliminary results are available for the measurement chain of the concentration of particulate matter, which refers to the monitoring of the air in a closed room for a time interval of about two hours, as described in Section 3.5. These results are summarized in Figure 13, where the blue line in the top chart is the concentration value of PM10 measured by the reference instrument APM-2 (sample interval 4 min), and the red line represents the average of groups of four readings (sample interval 1 min) of the sensor Sensirion SEN54. The bottom chart of the figure shows the measurement error of the device under verification with respect to the reference instrument (blue line).

The sensor was compliant with its stated specifications (the couple of red lines in the figure), and the obtained error is within the required uncertainty (the couple of black lines in the figure), even though the performed test refers to a small fraction of the concentration range of interest, which is $100\;\mu g/m^{3}$.

Further tests are planned to verify the metrological performance of the sensor under verification in the whole range of interest, also including the measurement of the concentration of PM2.5, thus obtaining a full characterization with respect to the measurement requirements.

### 4.6. Formaldehyde

**Figure 14:**
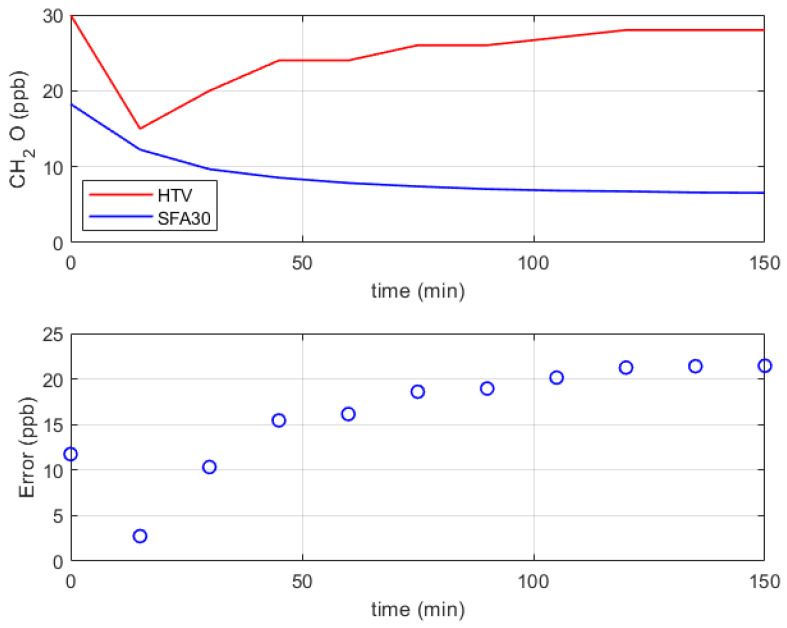
Experimental results related to the verification of the sensor Sensirion SFA30 for formaldehyde concentration.

Measurement with the formaldehyde low-cost sensor and the reference instrument lasted 150 min, with a brief waiting period before sampling to minimize any potential disturbances caused by air movements. For this reason, a particular trend can be seen in the first few minutes of the measurement, as shown in Figure 14. After that, the trends tend to stabilize over time. In the ambient atmosphere, formaldehyde exists within a diverse array of trace gases. In Figure 14, an acceptable difference is shown between the two measurements (Sensirion SFA30 and Formaldemeter htV-M), considering that the uncertainty stated by the sensor manufacturer is ±20 ppb and the difficulties in measuring formaldehyde at low concentrations. However, our comprehension of sensor performance is currently at an early stage. There is potential for the development of more advanced models to assess sensor performance in ambient conditions. It is worth noting that specific accuracy criteria for low-cost formaldehyde sensors have yet to be established [39].

## 5. Discussion

Performing continuous monitoring to assess IEQ is increasingly proving to be important, also due to the large amount of time that people spend in closed spaces [1]. In this study, the development and metrological characterization of the low-cost multi-sensor device that is part of the PROMET&O system are described. The PROMET&O system also includes a questionnaire for the collection of occupants’ feedback on comfort perception and a dashboard for data displaying to end users. The purpose of this system is to correlate objective and subjective data, provide information for more conscious management and use of building systems, and provide users with suggestions and more information on the topic of IEQ, promoting their proactive behavior to improve the environmental quality and their comfort perception.

The multi-sensor, which was designed for monitoring thermal, acoustic, visual, and air-quality conditions of indoor environments, embeds low-cost sensors that were selected to meet the identified requirements in terms of measurement range, uncertainty, physical dimensions, response time, and power consumption. Such sensors monitor air temperature (T), relative humidity (RH), illuminance (E*_V_*), sound pressure level (SPL), carbon monoxide (CO), carbon dioxide (CO_2_), nitrogen dioxide (NO_2_), particulate matter (PM), volatile organic compounds (VOC), and formaldehyde (CH_2_O). During the multi-sensor design phase, particular attention was given to the placement of hardware components to minimize mutual influences. The output signals of the sensors are connected to the onboard microcontroller that acquires data and, through a WiFi module, transmits them to a cloud server, where data are stored and post-processed to be then visualized in the web interface.

The highlight of this project is the reliability of the measured data by means of the implementation of a metrological confirmation process [25], which consists of identifying the requirements of the monitored parameters in terms of range and uncertainty and then characterizing each measurement chain to check its conformity with respect to the identified requirements. To this aim, commonly employed or ad hoc developed calibration procedures, based on the comparison to reference standards, were defined. At present, a calibration procedure has been defined for the bare sensors of T, RH, E*_V_*, SPL, CO_2_, PM and CH_2_O. The stated uncertainty was verified with respect to the required uncertainty in the measurement range of interest.

Many difficulties in calibrating sensors for air-quality monitoring were found, and in the literature, evidence is given. In relation to formaldehyde and carbon monoxide sensors, Parkinson et al. [21] stated that the calibration brings results adequate to detect problems related to compliant or not compliant values even though not sufficient for detailed investigations. Tiele et al. [18] also developed a multi-sensor device for continuous monitoring of IEQ conditions and not for safety-critical applications, which does not require rigorous testing.

With respect to other multi-sensors recently referenced in the literature [18,19,21], they all performed calibrations in the laboratory and/or in the field. In particular, refs. [18,21] performed laboratory calibration, whereas [19] did a second step test in the office environment. Within the PROMET&O project, it was planned to have a three-step calibration process which includes the calibration of the bare sensors in the laboratory, as described in this study, the calibration of the assembled multi-sensor in the laboratory, and the in-field evaluation in a controlled office against laboratory-grade sensors. In this way, the calibration process is based on continuous adjustments and upgrades, ensuring measurement traceability.

## 6. Conclusions

In this study, the metrological characterization of the bare sensors of the PROMET&O multi-sensor device is described, which was developed to continuously monitor indoor IEQ conditions. The main results of the calibration procedures are reported below.

- The calibration procedure of temperature and relative humidity sensors consists of a verification of the whole measurement chains against reference standards, obtaining errors that comply with the target maximum admitted errors.
- The measurement chains of illuminance, sound pressure level, and carbon dioxide required an adjustment to be performed since the results of the verification procedure did not meet manufacturer specifications. The adjustment procedure consists of modifying the calibration function of each measurement chain, thus compensating for offset and gain errors, and implementing the identified calibration functions in the PROMET&O system firmware.
- As far as the microphone is concerned, it was positioned on the top of the PROMET&O device to distance it as much as possible from the particulate matter sensor, which is the only hardware component that is equipped with a fan. The microphone inlet was oriented upwards, ensuring that the fan noise, which is approximately 24 dB(A) at 0.2 m as declared by the manufacturer, is expected to be negligible compared to typical office noise levels.
- Concerning the particulate matter and formaldehyde sensors, measurements were done only in baseline conditions that did not allow a full verification to be assessed. Thus, only preliminary results are provided.

The next steps that this study will include are summarized below.

- Further tests for particulate matter and formaldehyde sensors to verify their metrological performance in the whole range of interest, thus obtaining a full characterization with respect to the measurement requirements.
- The calibration of the sensors not included in this work (carbon monoxide, nitrogen dioxide, volatile organic compounds).
- The calibration of the assembled multi-sensor in the laboratory, with the aim of investigating the mutual effects of hardware components and refining the calibration functions.
- The in-field evaluation in a controlled office against laboratory-grade sensors, with the aim of assessing the uncertainty over the measurement ranges typical of offices.

## Figures and Tables

**Table 1:** Monitored parameters and threshold values in office environments.

Parameter	Threshold	Reference
Temperature (T)	Winter: (20–24) °C Summer: (23–26) °C	ISO 7730:2005 [10]
Relative Humidity (RH)	(30–70)%	ISO 7730:2005 [10]
Illuminance (E_v_)—writing, typing, reading, data processing	≥500 lx	EN 12464-1:2021 [13]
Carbon dioxide ($\Delta {\mathrm{CO}}_{2}$)	≤800 ppm	EN 16798-1:2019 [14]
Carbon monoxide (CO)	15 min. mean $\leq 100\;\mathrm{mg}/m^{3}$ 1 h mean $\leq 35\;\mathrm{mg}/m^{3}$ 8 h mean $\leq 10\;\mathrm{mg}/m^{3}$ 24 h mean $\leq 7\;\mathrm{mg}/m^{3}$	EN 16798-1:2019 (WHO guidelines) [14]
Nitrogen dioxide (${\mathrm{NO}}_{2}$)	1 h mean ≤200μg/m^3^ Annual mean ≤20μg/m^3^	EN 16798-1:2019 (WHO guidelines) [14]
Particulate matter (PM2.5)	24 h mean ≤25μg/m^3^ Annual mean ≤10μg/m^3^	EN 16798-1:2019 (WHO guidelines) [14]
Particulate matter (PM10)	24 h mean ≤50μg/m^3^ Annual mean ≤20μg/m^3^	EN 16798-1:2019 (WHO guidelines) [14]
Formaldehyde (${\mathrm{CH}}_{2}O$)	30 min. mean ≤100μg/m^3^	EN 16798-1:2019 (WHO guidelines) [14]
Volatile Organic Compounds (VOC)	≤500 μg/m^3^	WELL v2 [28]
Sound Pressure Level (SPL)	≤45 dB(A)	NF S 31-080 [12]

**Table 2:** Main characteristics of the selected commercial sensors for each monitored Parameter (P): Sensor Measurement Range (SMR), Declared Uncertainty (DU), and Range of Declared Uncertainty (RDU) by the manufacturer. The Required Uncertainty (RU) by standards and building certification schemes is defined for each parameter. The italic text is used to indicate sensors that are nominally able to meet the uncertainty requirement; the bold text is used to indicate sensors that require metrological characterization; the others have not been characterized yet.

Parameter (P)	Sensor Measurement Range (SMR)	Declared Uncertainty (DU)	Range of Declared Uncertainty (RDU)	Required Uncertainty (RU)	Reference
*Air temperature (T)*	(−40 to +125) °C	±0.2 °C	(0–60) °C	±0.5 °C	ISO 7726:2001 [29]
*Relative Humidity (RH)*	(0–100)%	±1.8%	(30–70)%	±5%	ANSI/ASHRAE 55:2020 [9]
**Illuminance (E_***V***_)**	(0–120) klx	15% mv	-	±5% at values ≤ 2000 lx	WELL, Q4 2023 [30]
Carbon monoxide (CO)	(0–1000) ppm	±2.75 nA/ppm (sensitivity)	-	±1 ppm at values between 0 and 10 ppm	WELL, Q4 2023 [30]
**Carbon dioxide (CO_2_)**	(0–40,000) ppm	±(30 ppm + 3% mv)	(400–10,000) ppm	±50 ppm + 5% mv at values between 400 and 2000 ppm	WELL, Q4 2023 [30]
Nitrogen dioxide (NO_2_)	(0–5) ppm	±30% mv	(0–5) ppm	±20 ppb at values between 0 and 100 ppb	WELL, Q4 2023 [30]
Particulate matter (PM2.5)	(0–1000) μg/m^3^	±(5 μg/m^3^ + 5% mv)	(0–100) μg/m^3^	±5 μg/m^3^ + 20% at values between 1 and 100 μg/m^3^	WELL, Q4 2023 [30]
Particulate matter (PM10)	(0–1000) μg/m^3^	±(25 μg/m^3^)	(0–100) μg/m^3^	±5 μg/m^3^ + 20% at values between 1 and 100 μg/m^3^	WELL, Q4 2023 [30]
Formaldehyde (CH_2_O)	(0–1) ppm	±20 ppb or ±20% mv, whichever is larger	(0–200) ppb	20 ppb at values between 0 and 100 ppb	WELL, Q4 2023 [30]
Volatile Organic Compounds (VOC)	(0–10,000) μg/m^3^	not declared	-	±20 μg/m^3^ + 15% mv at values between 1 and 500 μg/m^3^	WELL, Q4 2023 [30]
**Sound Pressure Level (SPL)**	122.5 dB(SPL) AOP	not declared	-	±0.5 dB (1 kHz)	WELL, Q4 2023 [30]

## Data Availability

Data available on request from the authors.

## Abbreviations

The following abbreviations are used in this manuscript:

ADC	Analog-to-Digital Converter
AOP	Acoustic Overload Point
BACS	Building Automation and Control Systems
BREEAM	Building Research Establishment Environmental Assessment Method
CH_2_O	Formaldehyde
CO_2_	Carbon Dioxide
DQO	Data Quality Objectives
DU	Declared Uncertainty
E_*v*_	Illuminance
IAQ	Indoor Air Quality
IBEM	Intelligent Built Environment Monitor
IEQ	Indoor Environmental Quality
IoT	Internet of Things
LED	Light Emitting Diode
LEED	Leadership in Energy and Environmental Design
MEMS	Micro-Electro-Mechanical Systems
MQTT	Message Queuing Telemetry Transport
NDIR	Non-Dispersive Infrared
NO	Nitrogen Monoxide
NO_2_	Nitrogen Dioxide
O_3_	Ozone
OPC	Optical Particle Counter
P	Parameters
PM	Particulate Matter
PMMA	Polymethyl Methacrylate
PA 11	Polyamide 11
PROMET&O	PROactive Monitoring for indoor EnvironmenTal quality & cOmfort
RDU	Range of Declared Uncertainty
RH	Relative Humidity
RU	Required Uncertainty
SAMBA	Sentient Ambient Monitoring of Buildings in Australia
SBS	Sick Building Syndrome
SMR	Sensor Measurement Range
SO_2_	Sulfur Dioxide
SPL	Sound Pressure Level
T	air temperature
TVOC	Total Volatile Organic Compounds
WHO	World Health Organization

## References

[1] Bluyssen P.M. (2020). Towards an integrated analysis of the indoor environmental factors and its effects on occupants. Intell. Build. Int..

[2] horr Y.A., Arif M., Katafygiotou M., Mazroei A., Kaushik A., Elsarrag E. (2016). Impact of indoor environmental quality on occupant well-being and comfort: A review of the literature. Int. J. Sustain. Built Environ..

[3] Ghaffarianhoseini A., AlWaer H., Omrany H., Ghaffarianhoseini A., Alalouch C., Clements-Croome D., Tookey J. (2018). Sick building syndrome: Are we doing enough?. Archit. Sci. Rev..

[4] Schweiker M., Ampatzi E., Andargie M.S., Andersen R.K., Azar E., Barthelmes V.M., Berger C., Bourikas L., Carlucci S., Chinazzo G. (2020). Review of multi-domain approaches to indoor environmental perception and behaviour. Build. Environ..

[5] Roskams M.J., Haynes B.P. (2021). Testing the relationship between objective indoor environment quality and subjective experiences of comfort. Build. Res. Inf..

[6] Wargocki P., Wei W., Bendžalová J., Espigares-Correa C., Gerard C., Greslou O., Rivallain M., Sesana M.M., Olesen B.W., Zirngibl J. (2021). TAIL, a new scheme for rating indoor environmental quality in offices and hotels undergoing deep energy renovation (EU ALDREN project). Energy Build..

[7] Levin H. (2006). Editorial indoor environmental quality. Build. Res. Inf..

[8] Malmqvist T. (2008). Environmental rating methods: Selecting indoor environmental quality (IEQ) aspects and indicators. Build. Res. Inf..

[9] (2020). Thermal Environmental Conditions for Human Occupancy.

[10] (2005). Ergonomics of the Thermal Environment—Analytical Determination and Interpretation of Thermal Comfort Using Calculation of the PMV and PPD Indices and Local Thermal Comfort Criteria.

[11] (2022). Acoustics—Measurement of Room Acoustic Parameters. Part 3: Open Plan Offices.

[12] (2006). Acoustics—Offices and Associated Areas—Acoustic Performance Levels and Criteria by Type of Area.

[13] (2021). Light and Lighting—Lighting of Work Places. Part 1: IndoorWork Places.

[14] (2019). Energy Performance of Buildings—Ventilation for Buildings. Part 1: Indoor Environmental Input Parameters for Design and Assessment of Energy Performance of Buildings Addressing Indoor Air Quality, Thermal Environment, Lighting and Acoustics.

[15] Parkinson T., Parkinson A., de Dear R. (2019). Continuous IEQ monitoring system: Context and development. Build. Environ..

[16] Mattoni B., Guattari C., Evangelisti L., Bisegna F., Gori P., Asdrubali F. (2018). Critical review and methodological approach to evaluate the differences among international green building rating tools. Renew. Sustain. Energy Rev..

[17] Heinzerling D., Schiavon S., Webster T., Arens E. (2013). Indoor environmental quality assessment models: A literature review and a proposed weighting and classification scheme. Build. Environ..

[18] Tiele A., Esfahani S., Covington J. (2018). Design and development of a low-cost, portable monitoring device for indoor environment quality. J. Sens..

[19] Geng Y., Zhang Z., Yu J., Chen H., Zhou H., Lin B., Zhuang W. (2022). An Intelligent IEQ Monitoring and Feedback System: Development and Applications. Engineering.

[20] García M.R., Spinazzé A., Branco P.T., Borghi F., Villena G., Cattaneo A., Gilio A.D., Mihucz V.G., Álvarez E.G., Lopes S.I. (2022). Review of low-cost sensors for indoor air quality: Features and applications. Appl. Spectrosc. Rev..

[21] Parkinson T., Parkinson A., de Dear R. (2019). Continuous IEQ monitoring system: Performance specifications and thermal comfort classification. Build. Environ..

[22] Astolfi A., Carullo A., Fissore V., Puglisi G.E., Arcamone G., Shtrepi L., Raviola E., Barbaro A., Espinosa G.R., Chiavassa P. Development and Metrological Characterization of a Multi-sensor Device for Indoor Environmental Quality (IEQ) monitoring. Proceedings of the 2023 IEEE International Workshop on Metrology for Living Environment (MetroLivEnv).

[23] Fissore V., Barbaro A., Chiavassa P., Espinosa G.R., Puglisi G., Raviola E., Giusto E., Shtrepi L., Servetti A., Montrucchio B. Development of a Multi-sensor Device for Indoor Environmental Quality Assessment. Proceedings of the Forum Acusticum 2023.

[24] Fissore V.I., Saugo M., Arcamone G., Puglisi G.E., Shtrepi L., Sassoli N., Cassone V.I., Paduos S., Corrado V., Servetti A. Definition of a methodology for occupants’ feedback collection on perceived indoor environmental comfort. Proceedings of the Healthy Buildings Europe 2023.

[25] (2003). Measurement Management Systems—Requirements for Measurement Processes and Measuring Equipment.

[26] Wei W., Wargocki P., Zirngibl J., Bendžalová J., Mandin C. (2020). Review of parameters used to assess the quality of the indoor environment in Green Building certification schemes for offices and hotels. Energy Build..

[27] Fissore V.I., Fasano S., Puglisi G.E., Shtrepi L., Astolfi A. (2023). Indoor Environmental Quality and Comfort in Offices: A Review. Buildings.

[28] (2018). The WELL Building Standard v2.

[29] (2001). Ergonomics of the Thermal Environment.

[30] (2023). WELL Performance Verification Guidebook, Q4, 2023.

[31] Zawawi S., Hamzah A., Majlis B., Mohd-Yasin F. (2020). A Review of MEMS Capacitive Microphones. Micromachines. Micromachines.

[32] LMP91051 NDIR CO2 Gas Detection System. https://www.ti.com/lit/an/snaa207/snaa207.pdf.

[33] Datasheet SEN5x. https://sensirion.com/resource/datasheet/sen5x.

[34] Directive 2008/50/EC of the European Parliament and of the Council of 21 May 2008 on Ambient Air Quality and Cleaner Air for Europe Europe. https://eur-lex.europa.eu/eli/dir/2008/50/oj.

[35] (2021). Air Quality—Performance Evaluation of Air Quality Sensor Systems—Part 1: Gaseous Pollutants in Ambient Air.

[36] Yatkin S., Gerboles M., Borowiak A., Davila S., Spinelle L., Bartonova A., Dauge F., Schneider P., Van Poppel M., Peters J. (2022). Modified Target Diagram to check compliance of low-cost sensors with the Data Quality Objectives of the European air quality directive. Atmos. Environ..

[37] (2017). Photometry—Part 7: Classification of Illuminance Meters and Luminance Meters.

[38] Yuan Z., Yang C., Meng F. (2021). Strategies for Improving the Sensing Performance of Semiconductor Gas Sensors for High-Performance Formaldehyde Detection: A Review. Chemosensors.

[39] Pei Z., Maxim Balitskiy R.T., Kelly K.E. (2023). Laboratory Performance Evaluation of a Low-Cost Electrochemical Formaldehyde Sensor. Sensors.

[40] Sun L., Wong K.C., Wei P., Ye S., Huang H., Yang F., Westerdahl D., Louie P.K.K., Luk C.W.Y., Ning Z. (2016). Development and Application of a next Generation Air Sensor Network for the Hong Kong Marathon 2015 Air Quality Monitoring. Sensors.

[41] Cross E.S., Williams L.R., Lewis D.K., Magoon G.R., Onasch T.B., Kaminsky M.L., Worsnop D.R., Jayne J.T. (2017). Use of Electrochemical Sensors for Measurement of Air Pollution: Correcting Interference Response and Validating Measurements. Atmos. Meas. Tech..

[42] (2015). Quality Management Systems.

[43] PPM Technology Ltd. (2018). PPM Formaldemeter^TM^ htV 3 Parameter IAQ Monitor.

